# Our Experience With 200 Cases of Inguinal Hernia Repair Using the Dynamic Self-Adjusting Prosthesis: A Case Series and Literature Review

**DOI:** 10.7759/cureus.68258

**Published:** 2024-08-30

**Authors:** Agostino Fernicola, Antonio Alvigi, Giovanni Angelone, Luigi Scotti, Alessandro Salvucci, Raffaele Finelli, Vincenza Capuozzo, Giovanni Aprea, Michele Santangelo, Giuseppe Scognamiglio

**Affiliations:** 1 Clinical Medicine and Surgery, Azienda Ospedaliera Universitaria Federico II, Naples, ITA; 2 General and Minimally Invasive Surgery, Ospedale S. Maria della Pietà dei Religiosi Camilliani, Casoria, ITA; 3 Advanced Biomedical Sciences, Azienda Ospedaliera Universitaria Federico II, Naples, ITA

**Keywords:** indirect inguinal hernia, direct inguinal hernia, inguinal hernia mesh fixation, open inguinal hernia repair, inguinal hernia complications, open surgery, inguinal canal, inguinal hernia repair, dynamic self-adjusting prosthesis, inguinal hernioplasty

## Abstract

Introduction

Inguinal hernioplasty (IH) is one of the most frequently performed surgical procedures globally. Today, a variety of surgical techniques and prosthesis types are available for this procedure.

Methods

At our center, we performed 200 inguinal hernioplasties using the dynamic self-adjusting prosthesis (protesi autoregolantesi dinamica*,* PAD) from May 1, 2022, to May 31, 2023. Our objective was to retrospectively analyze the outcomes and compare them with the current scientific literature on this surgical technique.

Results

Our results align with those reported by other authors using the same surgical technique. With the PAD technique, we assessed the type and frequency of adverse events up to 12 months following IH. All patients were male, with an average BMI of 26.6. Among the 200 hernias, 99 were right-sided, 101 were left-sided, 63 were direct, and 137 were indirect. The average length of hospitalization was one day. The most common postoperative complication was hematoma near the surgical site, but no prosthesis displacement was observed. In 71% of patients, analgesics were discontinued within 24 hours. The outcomes of our study are comparable to those reported by the inventor of this surgical technique.

Conclusion

The procedure has demonstrated safety and effectiveness and could serve as a viable alternative to traditional IH techniques.

## Introduction

The history of inguinal hernioplasty (IH) has spanned several centuries [1]. The first descriptions of IH date back to the 1st century with Celsus, followed by Galen, who described the inguinal canal (IC) for the first time. A habit of medieval surgeons was the recognition and sectioning of the spermatic cord. It took 700 years to achieve a postoperative hospital stay of a few hours. Since 1500, preservation of the testicle has become mandatory in all IH operations: the spermatic cord is dissected and the spermatic vessels of the sac are separated from the hernia sac. In 1884, Bassini sutured the conjoined tendon and the upper edge of the open transversalis fascia (triple layer) to the inguinal ligament. From Bassini onward, the problem has moved from the treatment of inguinal hernia to the prevention of recurrences, hence the advent of prostheses, primarily in steel, followed by marlex and mersilene. The first network was used by Uscher in 1958, followed by Rives, Stoppa, and Wantz. Currently, modern surgical techniques are based on the rational assumptions of the absence of tension in the hernial plastic (tension-free) and on the use of prostheses [2]. The tension-free technique derives from the evidence that the approximation of anatomical structures such as the inguinal ligament and the conjoined tendon is the cause of tension on the suture line. Tension on the suture line could cause rupture of the suture or tissue and therefore hernia recurrence. Lichtenstein was among the first to develop a tension-free hernioplasty technique [2]. The technique was later modified by Wantz, Trabucco, and Rutkow. The introduction of this technique has reduced the recurrence rate to below 1%. Recurrence can therefore be ascribed to the same biological, mechanical, and anatomical causes that are at the origin of primary hernias.

The most commonly used material in the construction of prostheses is monofilaments derived from nylon (prolene, polypropylene, or marlex) because it has shape memory [3,4]. The shapes and dimensions of prostheses can differ depending on the patient’s anatomy, which allows specific use of meshes [5,6]. Most networks are pre-shaped (the shape derives from the project of the author who proposed it) [6,7]. Meshes made of nonabsorbable material are the most used and vary based on the size of the pores of the prosthetic fabric [3,8]. Among the most commonly used non-absorbable materials is polypropylene [9]. The absorbable materials used are synthetic or biological (cadaveric dermis, submucosa and dermis of porcine intestine, and bovine pericardium) [10]. Over time, the biological material is replaced by the patient’s newly formed tissue. Biological prostheses are particularly indicated in cases of pediatric hernioplasty and in infected tissues [11-14].

There are three categories of inguinal hernia repair techniques [5,6,15-17]. The sublay technique involves positioning the mesh in the preperitoneal space via inguinotomy by opening the transversalis fascia (Rives, Pellissier), via a median longitudinal incision (Stoppa), via a lateral suprapubic transverse incision (Wantz), or via laparoscopic means creating a peritoneal flap (TAPP, TransAbdominal PrePeritoneal) or using a trocar remaining outside the peritoneal cavity (TEP, Total Extra Peritoneal) [18-20]. In TAPP, there is access to the abdominal cavity; in TEP there is no access to the abdominal cavity. In TEP, the space between the rectus muscles and the peritoneum is explored. The onlay technique involves positioning the mesh anterior to the transversalis fascia (Lichtenstein, Trabucco) [17,21]. The third category of technique involves the use of plugs inside the abdominal wall defect (Rutkov) [5,15,16].

According to the European Hernia Society, the traditional tension-free Lichtenstein technique is still defined as the gold standard for the treatment of primary unilateral inguinal hernia. Instead, for bilateral and recurrent unilateral inguinal hernias, the laparoscopic technique is preferred [22]. In Italy, the most commonly performed technique involves the positioning of the onlay prosthesis [1].

Protesi autoregolantesi dinamica (PAD) is a therapeutic tool for primary and recurrent inguinal hernias in adults [23]. PAD is made up of a double polypropylene mesh that must support the inguinal region in a complementary way to each other. Each of the two networks is anchored to a single side of the IC [24]. This arrangement of the two prostheses allows the PAD to adapt to the morphological variations of the IC when the patient changes the decubitus position from the clinostasm position to the orthostasm position and during movements. These characteristics allow its distension in the anatomical musculoaponeurotic planes [24]. The porosity of this material allows the development of neovascularized granulation tissue between its meshes. During its application, transparency allows control of the underlying structures.

Valenti et al. performed planimetry of the IC on 150 patients to determine the shape and dimensions of the two prostheses [23]. The angle between the inguinal ligament and the rectus abdominis muscle is approximately 55 degrees. This trapezoidal area can be strengthened using a prosthesis. The size of this area varies with the slope of the inguinal ligament. The inguinal ligament is approximately 1.6 cm from the internal inguinal ring [23]. In this area, the authors passed the oblique side of the maximum and minimum angles identified in the 150 patients. By superimposing the two maximum and minimum areas, an area common to both was identified. The lower prosthesis is placed first, followed by the upper prosthesis. The PAD is placed beneath the aponeurosis of the external oblique muscle with a digital maneuver. The lower prosthesis was designed in this common area [25]. The lower prosthesis has thus been made adaptable to all patients suffering from inguinal hernia. It has a trapezoidal shape, and on the medial side, it has a slot and an orifice for the passage of the spermatic cord. Its lateral side is free and rests on the inguinal ligament. The upper prosthesis was born from the same study on 150 patients used to design the lower prosthesis [25]. The upper prosthesis had a notch on the lateral side. This incision allows the spermatic cord to be superficial. Its medial side lies on the sheath of the rectus abdominis muscle.

When the patient moves from the clinostasm to the orthostasm, the relationships between the inguinal ligament, the external oblique muscle, the internal oblique muscle, and the transversus abdominis muscle change. Indeed, the distance between the rectus abdominis muscle and the inguinal ligament increases. Furthermore, the abdominal wall takes on a concave shape, and occasionally the lower portion of the abdominal wall may give way. In this latter case, it may be difficult to evaluate the right tension to give to the prosthetic material and the sutures [23]. The consequence is an increase in the relative risk of generating strings, tissue traction, and pain. In the PAD technique, Valenti et al. demonstrated a change in the relationships between the upper and lower prostheses marked with clips from the transition of patients from clinostasm to orthostasm to radiological control [23]. In fact, the lower prosthesis is medialized by 1.4 cm compared to the upper prosthesis. Furthermore, the lower denture descends approximately 2 cm during this movement. This medialization and caudalization of the lower prosthesis compared to the upper prosthesis demonstrates the independence and dynamism of the PAD [17]. Then, the spermatic cord passes between the two prostheses.

The upper prosthesis leaves no area of weakness due to the compensation given by the lower prosthesis [20,26]. At the end of the procedure, both prostheses must appear free of plications, well stretched, without tension and traction, and fit together and parallel to the inguinal floor [25]. Under no circumstances does a point of contact occur between the PAD and the preperitoneal space, reducing the related risks as in other techniques [5,6,16,20,25,26]. Thus, the preperitoneal space is never polluted by prosthesis material.

The primary objective of our study is to describe the PAD technique from a surgical point of view and report our results obtained after performing 200 inguinal hernioplasties on male patients in terms of postoperative complications. The secondary objective is to compare our results with those obtained by the surgeon who invented the PAD technique. with a review of the scientific literature regarding the same technique.

## Materials and methods

Patients’ selection and endpoint

**Figure 1:**
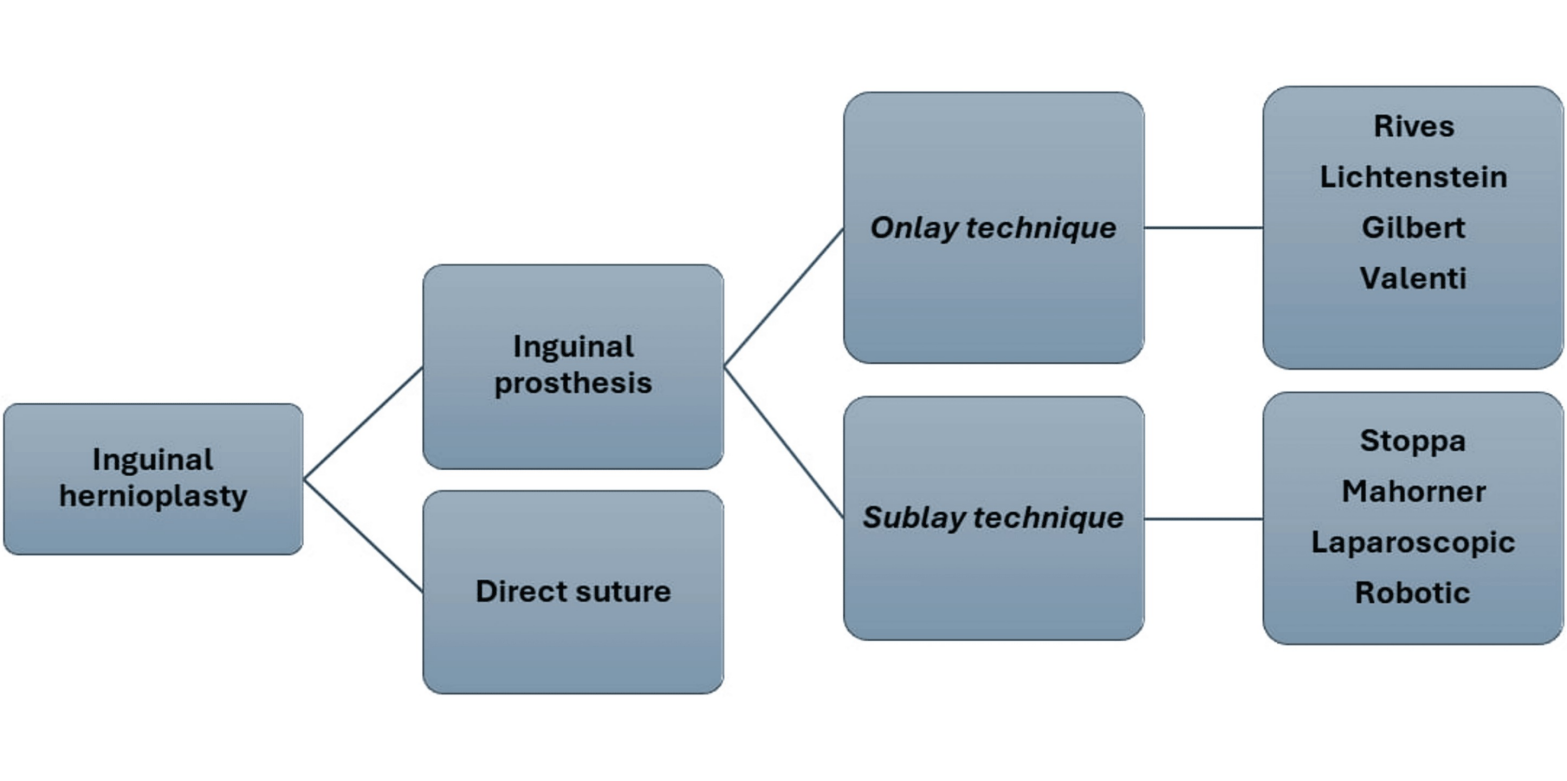
IH techniques for the treatment of inguinal hernia IH, inguinal hernioplasty

**Figure 2:**
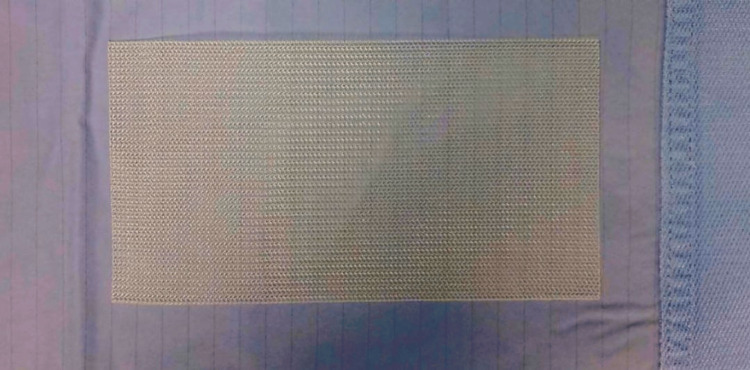
Polypropylene prosthesis from which the lower and upper prostheses are derived

**Figure 3:**
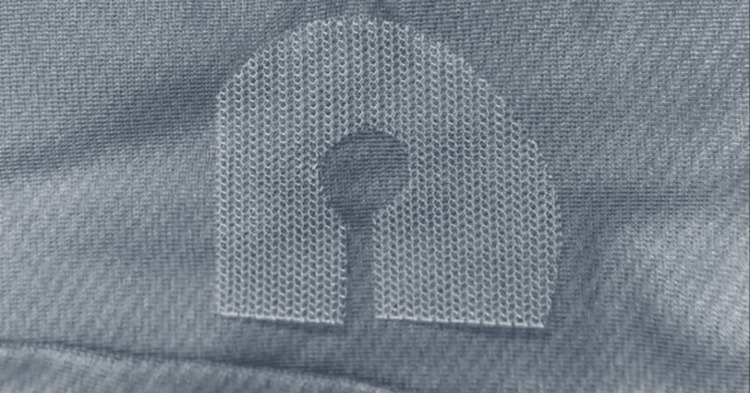
Lower prosthesis

**Figure 4:**
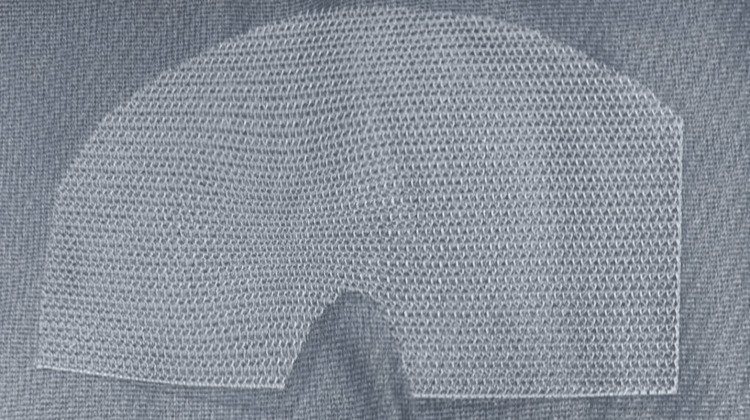
Upper prosthesis

**Figure 5:**
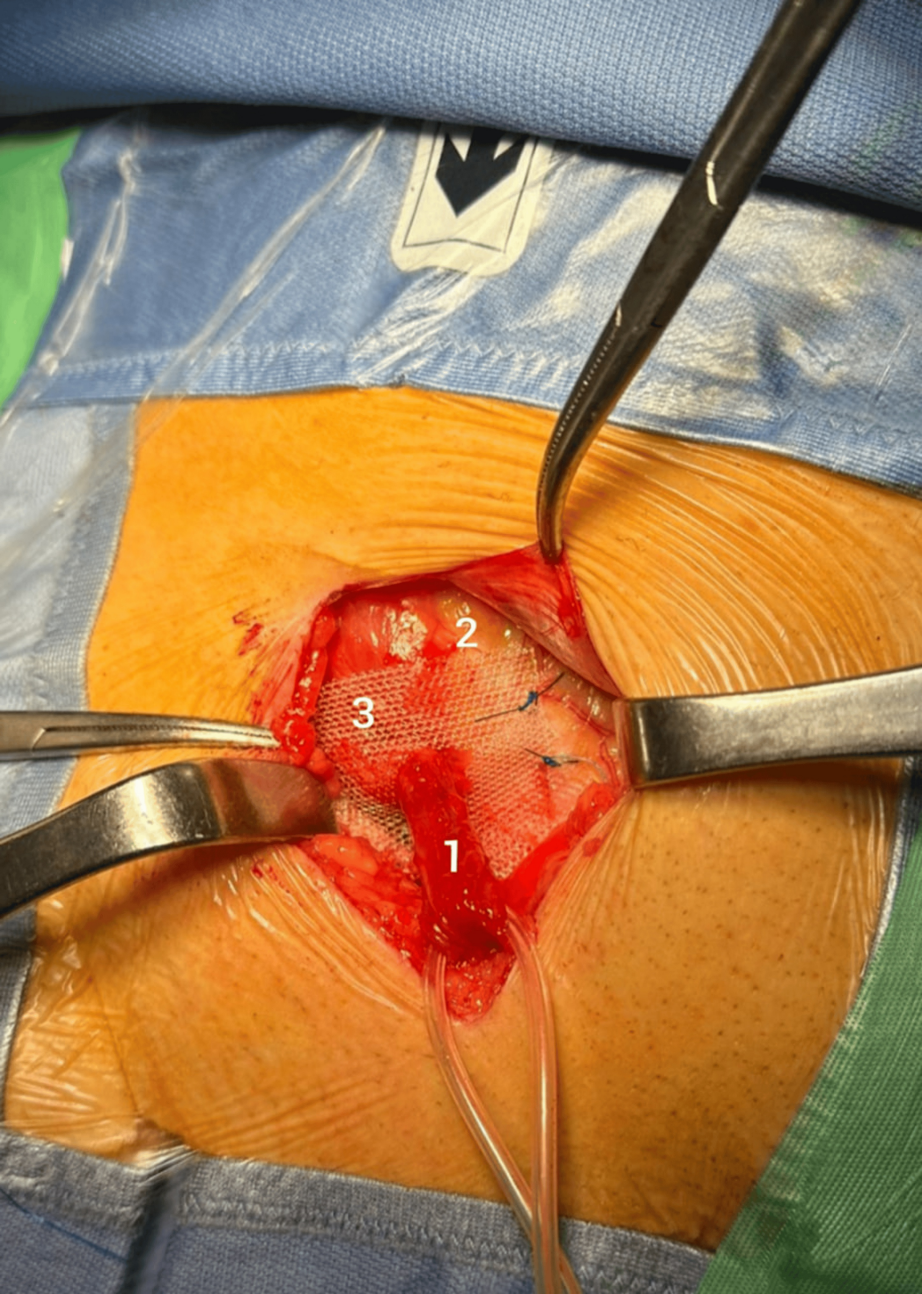
Lower prosthesis: (1) spermatic cord; (2) aponeurosis of the internal oblique muscle; (3) lower prosthesis

**Figure 6:**
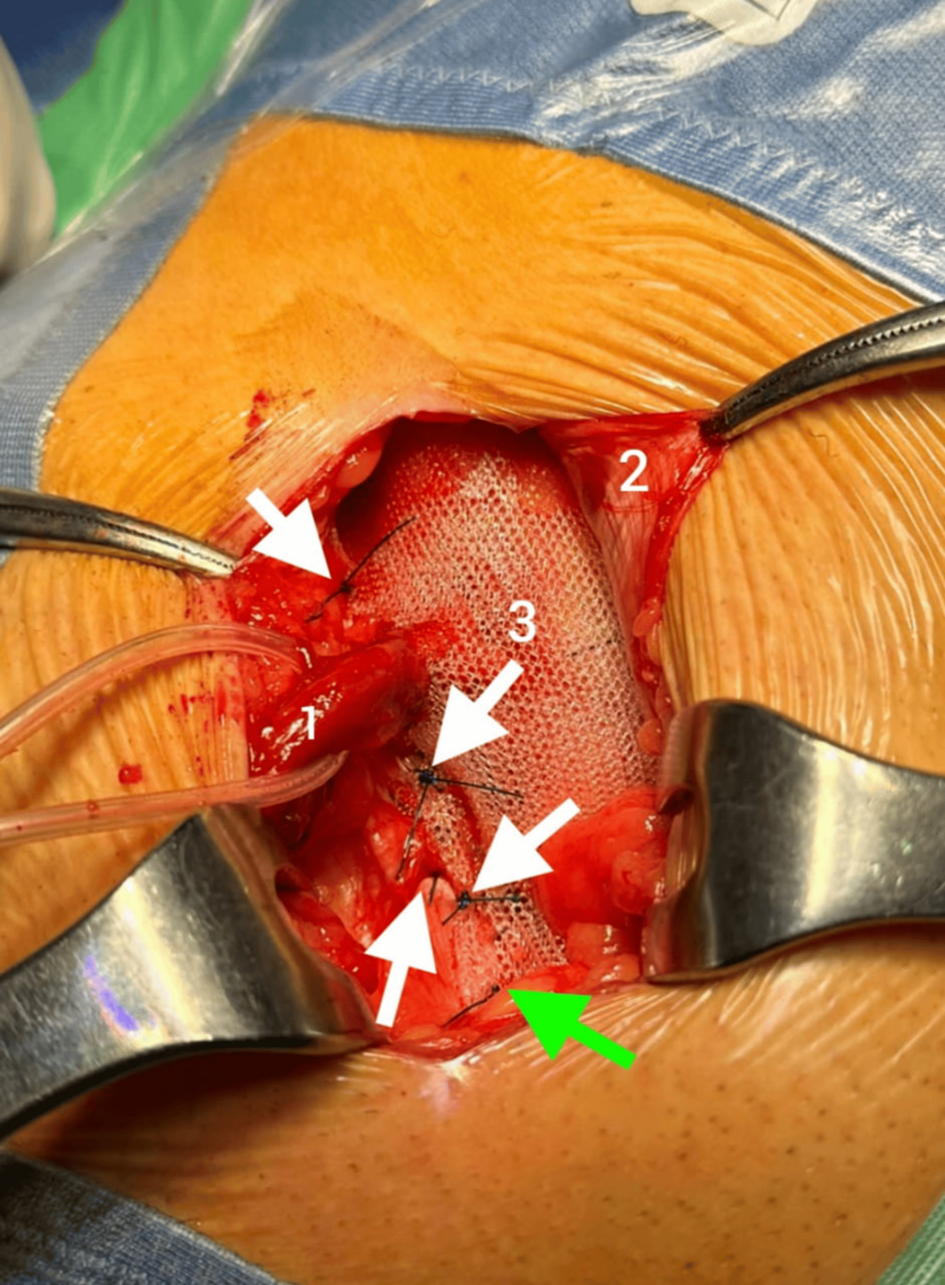
Upper prosthesis: (1) spermatic cord; (2) aponeurosis of the external oblique muscle; (3) upper prosthesis White arrow: the 4 anchoring points of the upper prosthesis on the inguinal ligament; green arrow: the anchoring point of the upper prosthesis on the pubic tubercle

We analyzed 200 consecutive inguinal hernioplasties performed on male patients between May 1, 2022 and May 31, 2023, in our General Surgery Department at the Santa Maria della Pietà dei Religiosi Camilliani Hospital in Casoria (Naples). All patients underwent laboratory and radiological examinations before surgery and received the same IH procedure with the same surgical technique used for PAD apposition (Figures 1-6). The surgeons who performed the inguinal hernioplasties all had at least 10 years of experience. We included unilateral (right or left side) hernias and reducible, irreducible, uncontainable, direct, indirect, recurrent, or nonrecurrent hernias. We excluded female patients because they were not included in the anatomical study proposed by Valenti for the creation of the PAD. Furthermore, we excluded all inguinal hernioplasties performed on an emergency or strangulated basis and cases of bilateral IH. Thus, out of a total of 278 patients, 200 male patients met the criteria of this retrospective study. All patients were aware of the surgical technique that would be used for IH. After providing written informed consent, patients were invited to visit the outpatient department for a physical examination 10 days and 12 months after surgery. Each patient was evaluated at the postoperative outpatient clinical checkup 10 days after discharge. In this context, we evaluated any postoperative complications such as hematomas, seromas, postoperative pain, inflammation, or surgical site infection. Furthermore, at the 12-month follow-up, we evaluated the presence of recurrence. If patients died during follow-up, all data were analyzed up to the time of death. Patients received a first-generation or second-generation cephalosporin-type antibiotic before surgery. For patients who were allergic to beta-lactams, we administered 600 mg of clindamycin or cotrimoxazole. Our aim was to retrospectively analyze the results obtained and compare them with existing scientific literature on the same surgical technique. This study is in accordance with the ethical standards of the institutional research committee (Santa Maria della Pietà dei Religiosi Camilliani Hospital), and we conducted this study after obtaining ethical approval from the Santa Maria della Pietà dei Religiosi Camilliani Hospital Institutional Review Board (IRB2022-001).

Materials and devices

Stitches of Prolene 0 and 2.0 and Vicryl 2.0 for the reconstruction of the structures of the IC (aponeurosis of the external and internal oblique muscles, transversalis fascia), Monocryl 2.0 was used for the reconstruction of the subcutaneous tissue, and Monocryl 3.0 was used for the creation of a continuous aesthetic skin suture. An electric scalpel and a cold blade scalpel were used for sectioning the anatomical structures of interest. Mayo and Metzenbaum scissors were utilized, along with Farabeuf and Mathieu retractors. Klemmer forceps were employed during the procedure. The dynamic self-adjusting prosthesis (PAD) was then applied.

Surgical procedure

After local anesthesia, we made an incision of approximately 5-6 cm between the iliac spine and the pubic tubercle. We dissected the skin and subcutis and opened Scarpa’s fascia. Subsequently, we identified the aponeurosis of the external oblique muscle and opened it with a cold blade scalpel along the direction of the fibers of the aponeurosis until the external inguinal ring was completely opened. Identification of the ilioinguinal and iliohypogastric nerves, blunt dissection of the aponeurosis of the external oblique muscle, and loading of the aponeurosis on Klemmer forceps were performed. We opened the cremaster muscle in its medial and lateral flap without sectioning it, recognizing the hernial sac and the structures of the spermatic cord and loading of the spermatic cord on Bottini forceps. Next, we identified the type of inguinal hernia. In the case of an indirect inguinal hernia sac, we reduced it into the internal inguinal ring, while in the case of a direct hernia sac, we prepared the posterior wall of the IC with a plication of the transversalis fascia with a continuous suture with absorbable monofilament. In the presence of a pre-hernial lipoma, we resected it at the base or sectioned it. In the case of a direct hernia sac, we always looked for the presence of a possible indirect hernia sac near the internal inguinal ring, visualizing the peritoneal reflection, and we always explored the crural canal to reduce the risk of future hernia recurrence. For the attachment of the PAD, we followed the standard procedure. Each of the two flaps of the lower prosthesis is sutured to the aponeurosis of the internal oblique muscle with a single stitch at the point where this aponeurosis merges with the anterior sheath of the rectus abdominis muscle [26]. The spermatic cord therefore acts as a stabilizer of the prosthesis [26]. The upper prosthesis is anchored with a single point at the pubic tubercle and with four detached points between its lateral side and the inguinal ligament. These last four detached points lateralize the spermatic cord and have no containment function; therefore, the inguinal cord will never be strangled. These stitches hold the spermatic cord in place and stabilize the lateral side of the lower denture. The fixation of the medial side of the upper prosthesis is achieved by suturing the lower prosthesis and is ensured between the sheath of the rectus abdominis muscle and the aponeurosis of the external oblique muscle after the latter is sutured [27]. The portion of the upper prosthesis positioned in the suprapubic position is exuberant to be positioned below the medial insertion tendon of the aponeurosis of the external oblique muscle. In this way, the external oblique aponeurosis acts to stabilize the PAD [27]. After the apposition of the PAD, we closed the aponeurosis of the external oblique muscle with a continuous suture and finally subcutis and skin with detached stitches or with an aesthetic intradermal suture.

Data collection

The clinical and pathological data of these 200 patients regarding the benefits and possible harms of this surgical technique were retrieved from the patient’s digital medical records and checked for accuracy. Data regarding age, sex, BMI, comorbidities, previous surgical interventions, drain placement, hernia type and laterality, average hospital stay, peri- and postoperative complications, and surgical duration were extracted from the patient’s electronic records. Patients were asked to categorize any complaints as “discomfort” or “pain.” The discomfort was defined as any unpleasant but nonpainful sensation that “irritated” or “bothered” the patient. “Chronic pain” was defined as any form of pain present three months after surgery, in accordance with international guidelines [28]. The results of this study are reported in line with the Preferred Reporting of CasE Series in Surgery guidelines [29]. The search for scientific articles regarding dynamic self-adjusting prosthesis (PAD) was conducted on PubMed, Web of Science, Scopus, and EMBASE using the following keywords: “dynamic self-adjusting prosthesis” AND “protesi autoregolantesi dinamica” OR “P. A. D.” OR “inguinal hernioplasty” OR “inguinal hernia repair” OR “hernia inguinal canal” OR “hernia open surgery.” We focused our attention on systematic reviews and observational studies on patient cohorts, randomized controlled trials, and meta-analyses. The included articles were either prospective or retrospective, monocentric or multicenter studies with a variable number of patients (hundreds to thousands). Only English studies were included.

Ethics statement

This study is in accordance with the ethical standards of the institutional research committee (Santa Maria della Pietà dei Religiosi Camilliani Hospital). We conducted this retrospective study after obtaining ethical approval from the Santa Maria della Pietà dei Religiosi Camilliani Hospital Institutional Review Board (IRB2022-001).

## Results

A total of 200 male patients who underwent IH surgery via the PAD technique were included in this retrospective study. All patients were male. The average age was 60.5 years. The average BMI was 26.8. The right/left laterality was basically equally proportioned. The most common comorbidity was arterial hypertension. The most common previous surgical interventions were IH and appendectomy. A total of 7% of cases involved IH for ipsilateral recurrence. In 13.5% of patients, we placed a drain. We placed a drain if we observed significant intraoperative bleeding or if there was a large inguinoscrotal hernia to reduce any risk of postoperative complications. The drain was always removed on the day of discharge. Inguinal hernias were predominantly indirect hernias. The average IH time was 40 minutes. Patients were able to walk three to four hours after IH surgery. The average in-hospital stay was eight hours; the majority of patients were discharged on the afternoon of the day on which the IH operation was carried out.

**Figure 7:**
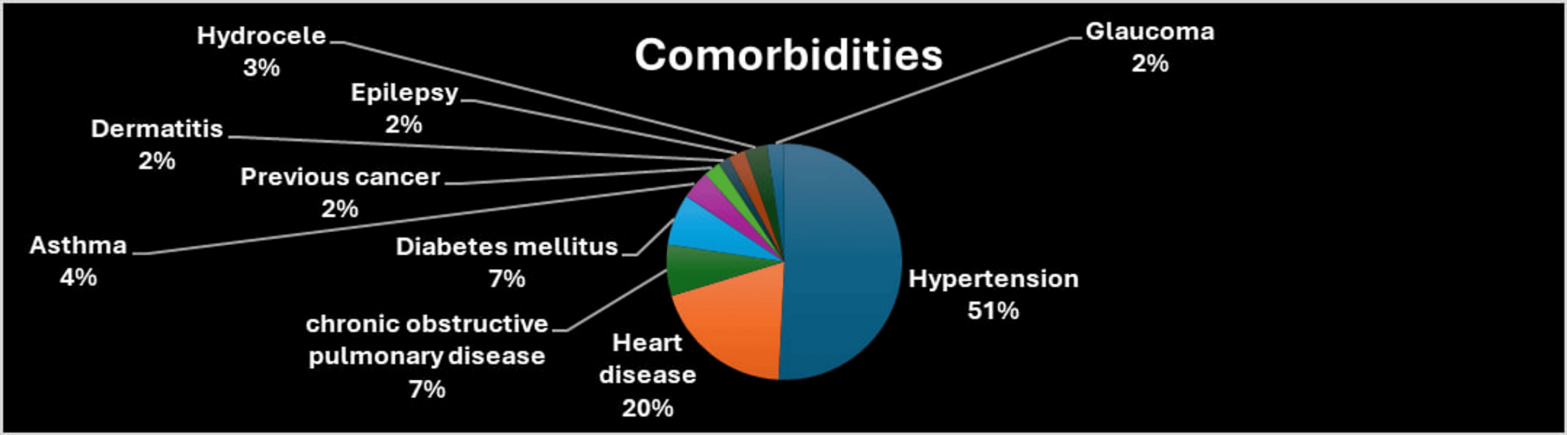
Preoperative comorbidities of patients

Table 1 summarizes the clinical and pathological features of the 200 patients. Table 2 summarizes the adverse events of IH with PAD at our hospital, and Table 3 summarizes the adverse events described by Valenti in his series of 585 patients. Figure 7 summarizes the main comorbidities of the patients.

**Table 1:** Clinical and pathological features of our 200 patients (2022-2023) n = absolute frequencies

Patients (number)	200
Age (arithmetic average)	60.5 years
Male (%)	100% (n = 100)
Female (%)	0% (n = 0)
BMI (arithmetic average)	26.6
Follow-up time (months)	12
Right inguinal hernia (number)	99
Left inguinal hernia (number)	101
Hernioplasties following previous recurrence (number)	15
Direct hernia (number)	63
Indirect hernia (number)	137
Days of hospitalization (arithmetic average)	1

**Table 2:** Adverse events in our experience (2022-2023) n = absolute frequencies

Hernias (number)	200
Postoperative mortality (%)	0% (n = 0)
Recurrences (%)	0.5% (n = 1)
Seromas (%)	0.5% (n = 1)
Hematomas (%)	1% (n = 2)
Orchites (%)	0% (n = 0)
Infections of the prosthesis (%)	0% (n = 0)
Wound infections (%)	0.5% (n = 1)
Displacement of the prosthesis (%)	0% (n = 0)
Displacement of the prosthesis (%)	0% (n = 0)
Discontinuation of the analgesic within 24 hours (%)	71% (n = 142)
Pain over four days (%)	2.5% (n = 5)
Persistent neuralgia beyond seven days (%)	0.15% (n = 3)

**Table 3:** Adverse events in Valenti’s experience (1992-1995) n = absolute frequencies

Hernias (number)	585
Postoperative mortality (%)	0% (n = 0)
Recurrences (%)	0% (n = 0)
Seromas (%)	0.5% (n = 3)
Hematomas (%)	0.34% (n = 2)
Orchites (%)	0% (n = 0)
Infections of the prosthesis (%)	0% (n = 0)
Wound infections (%)	0% (n = 0)
Displacement of the prosthesis (%)	0% (n = 0)
Displacement of the prosthesis (%)	0% (n = 0)
Discontinuation of the analgesic within 24 hours (%)	68.54% (n = 401)
Pain over four days (%)	5.12% (n = 30)
Persistent neuralgia beyond seven days (%)	0.34% (n = 2)

## Discussion

This retrospective study was conducted with the aim of evaluating the surgical technique proposed and patented by Gabriele Valenti, MD, called PAD during IH surgeries and comparing our results with those proposed by the author and with those presented in the scientific literature. In this retrospective study, we investigated the safety and feasibility of IH with a dynamic self-adjusting prosthesis (PAD). The use of PAD appeared feasible in all cases, and we never had to resort to other techniques. We observed a minimal complication rate associated with the surgical procedure, in line with the data present in the scientific literature.

Each surgeon develops his own experience using techniques that are often already known [5]. The examination of the results deriving from their application represents the stimulus to seek and propose new solutions to the scientific community [16]. For example, the problem of the tension of direct sutures led Lichtenstein to create a tension-free technique, standardizing the use of prosthetic material [6,16]. Subsequently, the twists, plications, and tensions caused by the use of a prosthetic mesh sutured around the perimeter led Gilbert and other authors to apply a prosthesis without stitches [17]. Another common limitation of prosthetic meshes is the need to cut out the shape and size for each operation, to fix the prosthesis or not, and to evaluate the different sizes [5,7,16,28]. All these variables make it difficult to standardize and simplify a surgical technique and often require personal evaluation by the surgeon, which increases the risk of making an error. For these reasons, Valenti et al. developed a surgical technique for IH indicated for primary, direct, and indirect inguinal hernias [23]. The dynamic self-adjusting prosthesis (in Italian, PAD) has the advantage of being made up of a double pre-shaped prosthetic complex made of polypropylene of a single standardized size, fixed to the anatomical structures but at the same time mobile [23,24]. In this way, the intent is to overcome the limits of a prosthesis fixed throughout its perimeter, of a free prosthesis, and of a prosthesis positioned preperitoneally [23]. The preparation of the IC is the same as that performed for an onlay IH operation [23,24]. In our experience, we can confirm in all patients the lower prosthetic layer can only move medially as it is fixed only medially and thus reduces the internal inguinal ring. The upper prosthetic layer always remains stationary without ever undergoing any traction, as it is only fixed laterally to the inguinal ligament. We observed that the lower prosthesis is stabilized by the spermatic cord and the lateral edge of the upper prosthesis. The upper prosthesis is stabilized by the spermatic cord and by pinching between the aponeurosis of the rectus abdominis muscles and the aponeurosis of the external oblique muscle. The two prostheses accommodate all the variations in relationships between the anatomical structures of the IC because they are separately anchored to opposing structures of the IC. Confirming the data presented in the literature, in our experience, in no case have we had to cut out the pre-packaged prostheses or take particular measurements of the IC [23-27]. The technique was easy to replicate. In our experience, we have also performed this technique even in cases of previous recurrence after IH. Our postoperative results conform to those primarily published by Valenti and subsequently by the authors who replicated the PAD technique. Although our results are in accordance with the literature and promising, we interpreted the results with caution. We are aware of the need for longer follow-ups of patients and to increase the number of patients studied in the future. The limits of our study are certainly the small sample size (200 patients) and the follow-up of only up to 12 months. Further retrospective studies are therefore necessary to confirm or refute the results obtained by our working group and those already present in the scientific literature.

## Conclusions

The IH technique with PAD is currently standardized. We found the technique easy to replicate and easy to perform, even in cases of large hernias. Under no circumstances did we have to cut out and modify the size and shape of the pre-shaped PAD, which therefore adapted well to the anatomy of the IC. Postoperative complications, both short and long term, were limited, similar to the results found in the scientific literature. This suggests that the PAD IH technique is a valid alternative to the open-onlay IH technique.
